# Implications of targeted next-generation sequencing for bladder cancer: report of four cases

**DOI:** 10.1186/s43141-021-00182-7

**Published:** 2021-06-21

**Authors:** Mohamed K. Khalifa, Noha M. Bakr, Amal Ramadan, Khaled M. Abd Elwahab, Esam Desoky, Amira M. Nageeb, Menha Swellam

**Affiliations:** 1 CSO at Omicsense, Cairo, Egypt; 2 Biochemistry Department, Genetic Engineering and Biotechnology Research Division, National Research Centre, El-Bohouth Street, Dokki, Giza, 12622 Egypt; 3 High Throughput Molecular and Genetic Laboratory, Center for Excellences for Advanced Sciences, National Research Centre, El-Bohouth Street, Dokki, Giza, 12622 Egypt; 4 Urology Department, Zagazig University, Zagazig, Egypt

**Keywords:** Bladder cancer, Somatic mutation, Germline mutation, *BRCA* genes, Next-generation sequencing, Case report, Treatment, Tumor suppressor genes, Sequencing

## Abstract

**Background:**

Bladder cancer is considered heterogeneous diseases with two major subgroups: non-muscle- invasive bladder cancer (NMIBC) and muscle invasive bladder cancer (MIBC). It is a major healthcare problem, and it is one of the leading causes of mortality worldwide. Genetic mutations are not only a cause for carcinogenesis but are also a way for treatment strategy. The present study aimed to investigate breast cancer (BRCA genes) tumor suppressor gene mutations in bladder cancer tissue and combined blood samples for patients who developed secondary tumor after or during trimodal therapy. Fresh tissue samples and their matched blood samples were collected from four patients with bladder cancer. The objective regions for the examined genes (*BRCA1* and *BRCA2*) were sequenced using next-generation sequencing (NGS); generated BAM files were uploaded to the cloud-based Ionreporter server, and the Oncomine BRCA-specific plugin was used to analyze the paired normal and tumor sample for each patient using the default plugin parameters.

**Results:**

Intronic BRCA1 mutation c.5050-104 C >T was reported among the four investigated bladder cancer patients, and three somatic mutations were reported as follows: two of them were found to be benign rs1064793056 and rs28897679 on the Clinivar database and one nonsense pathogenic variant rs80357006. BRCA 2 gene mutation reported an exonic synonymous mutation rs397507876 in the tissue and germline DNA. Patients were treated with trimodal; however, three bladder cancer patients who reported BRCA mutations developed secondary tumors.

**Conclusion:**

Identification of mutational BRCA changes in bladder cancer is a promising marker for better treatment strategy. Further studies are encouraged on a large cohort of bladder cancer patients to confirm our findings.

## Background

In developed countries, the frequency rate of bladder cancer is about 9.5/100,000; the most common type of it is urothelial carcinoma, which represents around more than 90% [1]. In Egypt, urinary bladder cancers represent 30% of all cancer cases with an incidence rate of 13.5/100,000 individuals according to the National Cancer Institute in Egypt [2]. The incidence of bladder cancer is growing with the advance of the economy, and its recurrence degree has developed a main economic burden on the health care systems [3]. The standardized mortality rate reported for bladder cancer was reported to be in males and females as 2–10/100,000 and 0.5–4/100000 per year, respectively, in 2016 [4]. Environmental factors, smoking, exposure to toxic industrial chemicals and gasses, and gene mutations of bladder cells are associated with increased incidence of bladder cancer [5].

Gene mutations play a significant role in the incidence and progress of bladder cancer. It is well known that genes such as fibroblast growth factor 3 (FGFR3), retinoblastoma (RB1), gene belong to RAS genes (HRAS), total p-53 (TP53), and hamartin (TSC1) can regulate the normal cell cleavage and avoid carcinogenesis. Hence, mutations in these genes can cause cancer [6]. In a previous study carried out in the USA, fifty-four cases with bladder cancer were sequenced and reported mutation in breast cancer-associated protein (BAP1) in about 15% of the enrolled cases which was an earliest attempt [7], which further directs to the alteration in breast cancer gene (BRCA) gene pathway and facilitates the induction for the features of papillary histological modification in bladder cancer [7]. Also, the relation between BRCA1 mRNA and cisplatin-based neoadjuvant chemotherapy was studied [8] and emphasized that the detection of BRCA1 mRNA expression could estimate both the sensitivity to chemotherapy and prognosis among patients with bladder cancer [8].

Structurally, the BRCA gene is complicated with slight information reported around it. Generally, it is the tumor suppressor gene and consists of two genes, i.e., BRCA1 and BRCA2; regarding its function, it has been reported that it has a role in DNA repairing mechanism, controlling the growth of the cell and blocking gene mutations, however, each with altered role on the targets. BRCA1 gene ended with an amino acid containing histone (H2AX) that is able to phosphorylate through infrared induction in a brief time, repairing DNA double stranded and assisting chromatin remodeling; hence, histone (H2AX) continues to be the main molecule for allowing BRCA1 gene to exert its function [9]. For BRCA2 gene, its repairing mechanism is done through RAD51 rather than H2AX. The regular expression of RAD51 certifies the repairing process through catalyzing the core reactions of homologous recombination (HR), with strand incursion into duplex DNA and the pairing of homologous DNA strands, thus allowing strand switch; on the other hand, overexpression or silencing of BRCA2 gene mutation leads to the collapse of the repairing process [10], causing gene error which results in the development of various tumors such as breast cancer [11], ovarian cancer [12], prostate cancer [13], and recently glioblastoma multiforme [14], but still, there is no information about the role of BRCA2 mutation in invasive urothelial bladder cancer [15].

The authors in this study aimed to investigate BRCA tumor suppressor gene mutations in bladder cancer-paired tissue and blood samples for patients who developed secondary tumor after or during trimodal therapy.

## Methods

After obtaining approval from the medical ethical committee, the current study was carried out on four patients with bladder cancer. The inclusion criteria for selected patients were based on patients with bladder cancer, and no other type of malignancies was reported; those did not fulfill these criteria were excluded. After obtaining informed consent, all patients underwent diagnostic cystoscopy (to evaluate tumor size site number and associated pathology), and transurethral resection of bladder tumor (TUBT) was done. Samples of fresh tissue of urinary bladder tumors and blood samples were obtained from each patient; their clinicopathological characteristics are reported in Table 1.

**Table 1:** Demographic and clinicopathological characteristics for bladder cancer cases

Characteristics	Case 1	Case 2	Case 3	Case 4
**Gender**	Male	Male	Male	Male
**Age**	65 years	53 years	47 years	57 years
**Family history of cancer**	Not reported	Not reported	Not reported	Not reported
**Primary tumor stage**	T1	T2	T2	T2
**Tumor grade**	G3	G3	G3	G3
**Final histopathology**	T2G3	T2G3	T2G3	T2G3
**Associated pathology**	Leukemia	LT renal RCC	Renal Hodgkin lymphoma	DM
**Management**	TURB	TURB and radical cystectomy with ileal conduit	Trimodal therapy	Trimodal therapy

Fresh tissue samples were collected and divided into two sections: one section was sent for pathological examination and stained with hematoxylin and eosin (H&E); the second tissue section was transferred to the lab in tubes with RNAse latter (storage reagent which allows tissues to stabilize and keep cellular RNA and reduces the need to immediately treat tissue samples or to freeze them in liquid nitrogen for further processing) for NGS processing. Paired blood samples were collected in EDTA containing tubes for further DNA extraction and NGS processing.

## Clinical evaluation

### Case #1

A male patient, 65 years old, presented with urinary bladder mass invading the left ureteric orifice with moderate backpressure on the left kidney, and the patient gave a history of two transurethral resections of bladder tumors (TURB) 1 year before their pathology was non-muscular invasive bladder cancer (NMIBC). The patient was hypertensive with no family history of bladder cancer, and serum creatinine was 5 mg/dl, total leukocyte count (TLC) 40 10^9^/L, and platelets count 1.2.10^9^/L. The patient underwent left nephrostomy for his renal insufficiency, bone marrow aspiration for leukocytosis, and thrombocytopenia Then, the patient was deteriorated as his bone marrow aspiration reported the presence of leukemia, and he died while being treated for leukemia.

### Case #2

A male patient, 53 years old, smoker presented with hematuria and blood clots; radiological investigations revealed a 5-cm bladder mass at the base of the bladder; the patient gave a past history of cystolithotomy, cholecystectomy, and HCV treatment, no history of chronic medical diseases, and there was no family history of the same illness. TURBT revealed a high-grade muscle invasive urothelial carcinoma. After 1 month, radical cystectomy with ileal conduit diversion was done, and its histopathology revealed T2G3 with negative lymph node involvement. The patient was followed up, and at 8 months of follow-up, he developed a 4-cm left renal lower pole mass, for which partial nephrectomy was done, and its histopathology revealed papillary renal cell carcinoma (RCC).

### Case #3

A male, 47 years old, heavy smoker presented to outpatient clinic with complaints of hematuria with blood clots and no chronic medical diseases (CMD); he had no family history of the same illness. The patient had a past history of internal fixation for left femur fracture. According to his imaging investigations, he was suffering from left bladder wall mass. TURBT was done, and its report revealed left lateral wall 4.5 cm mass high-grade transitional cell carcinoma (TCC) with muscle invasion. The patient was treated with trimodal therapy and followed up with CT with contrast. The patient responded to treatment, but his kidneys showed bilateral hypo-dense renal masses. Renal biopsy revealed Hodgkin lymphoma, and the patient was referred for chemotherapy.

### Case #4

A male patient, 57 years old, presented to the outpatient clinic with complaints of hematuria with blood clots and history of diabetes mellitus on insulin treatment and irrelevant family history. His radiology revealed multiple bladder masses; diagnostic cystoscopy revealed multiple bladder masses, and TURBT was done. Pathology report revealed high-grade transitional cell carcinoma (TCC) with muscle invasion. The patient was treated with trimodal therapy.

### Molecular analysis

#### DNA extraction

Genomic DNA was extracted from tissues and their matched blood samples using commercially available kits as follows: DNA extraction was done according to the manufacturer’s instructions of QIAamp DNA Mini blood kit (Cat No # 51104, Qiagen, Germany) based on spin column for DNA extraction method, while tissue DNA was extracted using Cat No # 51304, Qiagen, Germany from fresh tissue samples.

Both purity and the concentration for extracted DNA were detected by nano-drop spectrophotometer (Quawell, Q-500, Scribner, USA); then, extracted DNA was stored at − 80 °C till further assessments.

#### Library preparation and purification

The required regions for the examined genes BRCA1 and BRCA2 were amplified by Oncomine BRCA1 & BRCA2 research kit (Life Technologies). Amplification process was carried out by means of Ion AmpliSeq Library kit (Cat No# 4480441 Life technologies), according to the manufacturer’s protocol. After amplification, the primers were digested using FuPa Reagent; the samples were barcoded with Ion Xpress Barcode Adaptors. Barcoded libraries were then purified using Agencourt beads, and libraries were measured using the Ion Library TaqMan Quantitation kit (Cat No# 4468802 Life technologies). The quantified libraries were promoted to template preparation.

#### Template preparation

The obtained libraries from the previous step were pooled on molar equivalent rations to yield at least average 150x depth for coverage regarding every germline DNA sample and 750x for somatic samples. The assembled libraries were clonally amplified using Ion PGM Hi-Q view OT2 kit (Cat No# A29900 Life technologies) on the Ion OneTouch 2 instrument (Life technologies, USA) according to the manufacturer’s instructions. Then, the template ion sphere particles (ISP) were enriched using Ion PGM enrichment beads (Cat No# 4478525 Life technologies) by Ion OneTouch ES system (Life technologies, USA) according to the manufacturer’s instructions; the positive ISP Quality was assayed on Qubit 2.0 Fluorometer (Life technologies, USA) and then continued for accomplishing the sequencing process.

#### Sequencing using ion torrent PGM platform

Subsequently, calibrations and adjustments of the pH were done according to the manufacturer’s instructions using the Ion PGM Hi-Q View Sequencing kit (Cat No# A30044 Life technologies). Entirely barcoded enriched samples were sequenced on the Ion Torrent PGM Platform (Ion Torrent PGM, Life technologies, USA) using Ion 318 Chip Kit V2 BC (Cat No# 4488150 Life technologies).

#### Data analysis

Generated BAM files were uploaded to the cloud-based Ionreporter server version 5.10 on ThermoFisher website, and the Oncomine BRCA-specific plugin was used to analyze the paired normal and tumor sample for each patient using the default plugin parameters.

## Results

**Fig. 1:**
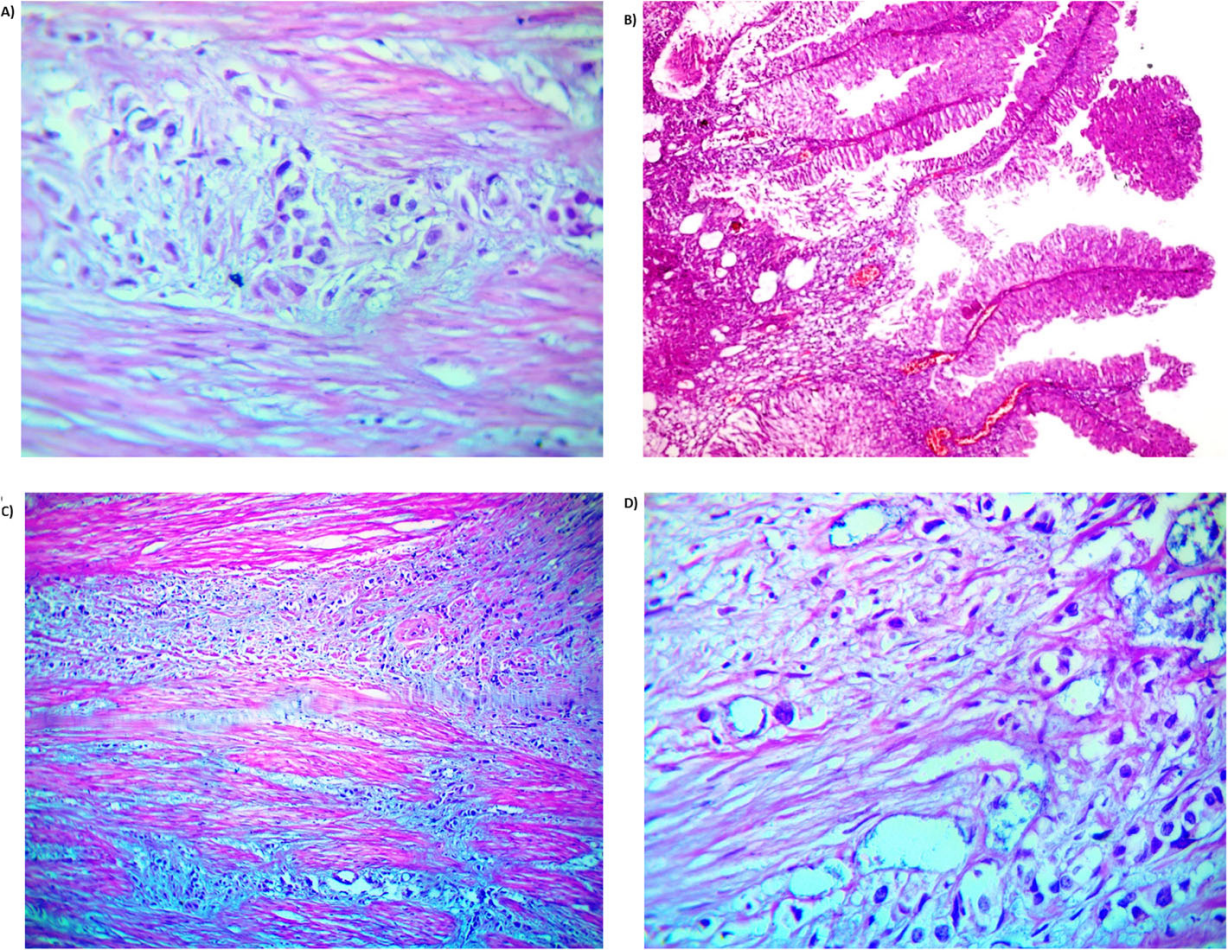
Cross section of urothelial bladder tissues stained with H&E. **a** High-grade urothelial carcinoma infiltrating deep muscle layer (pT2) (H&E × 400), **b** papillary urothelial neoplasm of high malignant potential (H&E × 200) (pTa), **c** high-grade urothelial carcinoma infiltrating deep muscle layer (pT2) (H&E × 200), and **d** high-grade urothelial carcinoma infiltrating deep muscle layer (pT2) (H&E × 400)

**Fig. 2:**
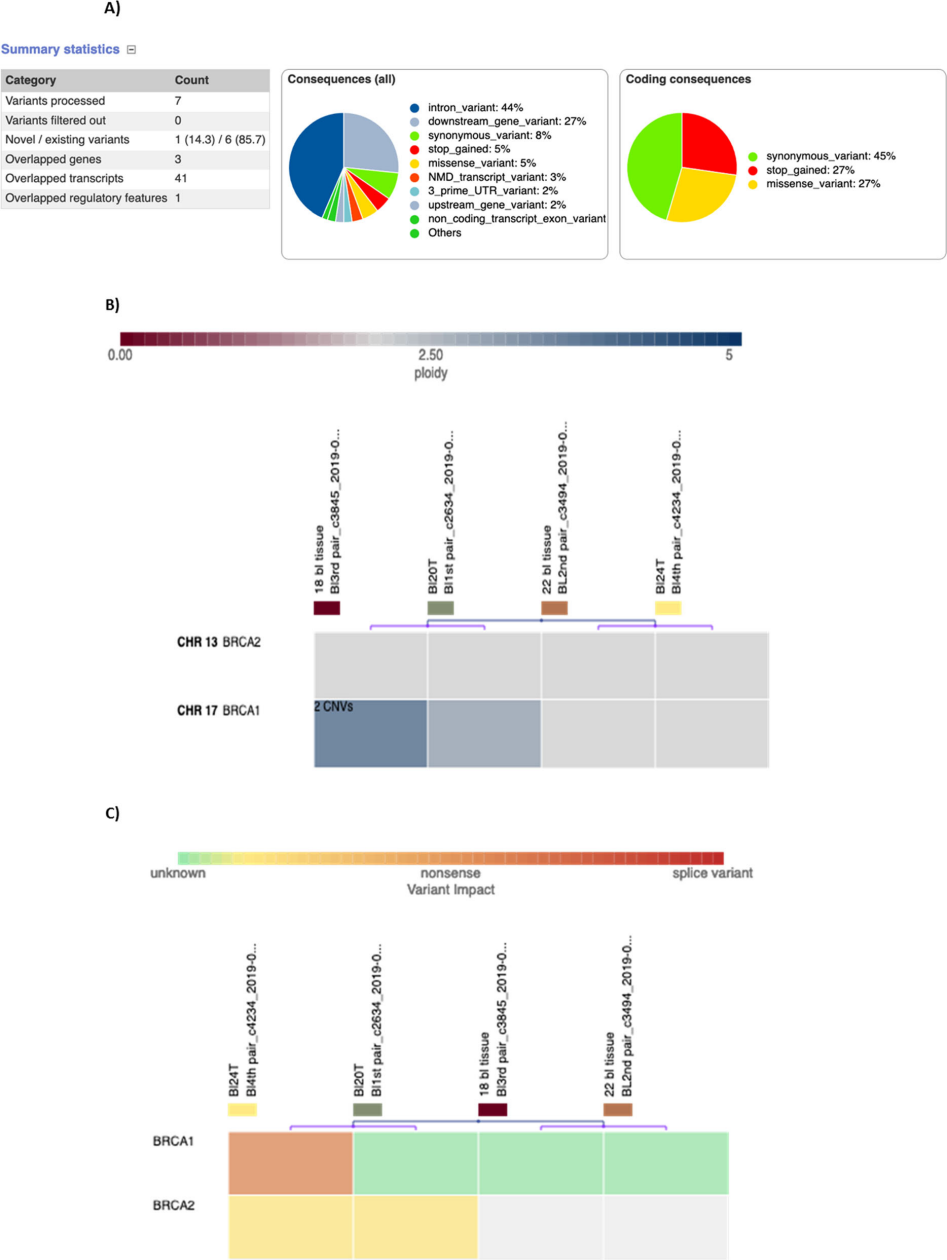
Analysis of NGS data report for the investigated samples. **a** Variant effect predictor diagram showing the detect variant effects in different BRCA transcripts. **b** Variant impact diagram showing the CNV heatmap. **c** Variant impact diagram showing the detected variants effects

Histopathological examination for the four enrolled samples was shown in Fig. 1a–d. Paired sample analyses for blood and tumor tissue were sequenced for *BRCA* genes (*BRCA*1 and 2). Analysis of NGS data output for investigated samples were presented in Fig. 2a–c; accordingly, each case were analyzed and reported as follows.

### Case #1

In case #1, there was an intronic *BRCA1* mutation c.5050-104C>T along with three copies for the 17q21.31 (41197601-41276123) region. Regarding *BRCA2*, there was an exonic synonymous mutation rs397507876 in the tissue and germline DNA, while there was no detectable difference between the copy numbers in BRCA2.

### Case #2

Only *BRCA1* gene reported intronic mutation at vc.5050-104C>T, without any copy number variations. For BRCA2, no genetic alteration in the copy number or in the sequences was reported.

### Case #3

In case #3, there was an intronic *BRCA*1 mutation c.5050-104C>T along with four copies for the 17q21.31 (41197601-41234616) and 17q21.31 (41249157-41276123) regions, while normal copy number for the rest of the gene was reported. Regarding *BRCA2*, there was no detectable difference between the copy number or structural variations between the blood and tissue.

### Case #4

Intronic *BRCA1* mutation c.5050-104C>T was reported combined with three somatic mutations; two of them were found to be benign rs1064793056 and rs28897679 on the Clinivar database, and one was nonsense pathogenic variant rs80357006 while copy number was normal. Regarding *BRCA2*, there were no detectable differences between the copy numbers; only one synonym mutation rs397507876 with an intronic variation rs2126042 was detected.

## Discussion

Bladder cancer is caused by many aspects. A number of genes perform an important role in the incidence and progression of the bladder cancer. The main mechanisms which result in bladder cancer are mutations of oncogenes and tumor suppressor genes [16]. Previous studies have exposed that *E2F3*, *MMP*, *FGFR3*, and *HER-2* genes played a stimulating role in the occurrence of bladder cancer [17], while *PTEN*, *p53*, *Rb*, *p27*, and *DMBT1* played an inhibitory task [18].

The status of DNA damage response (DDR) mutation of cancer is a significant predictive biomarker of immune checkpoint blockade (ICB) response [19]. Tumors with DDR mutations are immunologically hot and approachable to ICB. Clinical examinations on urothelial cancer, NCT02553642, NCT02108652, and NCT01928394 (www.clinicaltrials.gov), have established that mutations in DDR genes are predictors of response to PD-1/PD-L1 ICB [20]. Furthermore, it is documented that tumor cells with DDR shortage show constitutive triggering of cellular IFN responses and emission of TIL conscripting chemokines, CCL5 and CXCL10 [21, 22]. DDR mutation status of a cancer could be shared with complementary biomarker methods for patient selection for ICB [23].

The influence of *BRCA* genes generally lies in repair of DNA and preservation of gene stability. Previously, it was reported that the normal *BRCA* gene expression was of distinguished significance in the prevention of gene mutation such as *BRCA1*and *BRCA2* which plays a major role in the DDR pathway. Mutations in these two genes and intragenic copy number variation, which was reported as a source of pathogenicity in these genes [24], played a diagnostic and prognostic role in breast and ovarian cancers [25]. The current study reported pathogenic BRCA copy number variations and SNV in bladder tissues which emphasize their relevance in the pathogenesis of bladder cancer. In a recent study based on next-generation sequencing for bladder cancer patients who underwent transurethral resection and were treated with bacillus Calmette–Guérin (BCG) revealed the detection of some potential biomarkers and therapeutic targets and that DDR genes alterations were correlated with high tumor burden [26].

Trimodal treatment is one of the choices for bladder cancer patients; it comprises maximal transurethral resection of bladder tumors (TURBT) followed by altered regimens of combined radio and chemotherapy which achieved comparable results to radical cystectomy in many trials [27]. It has been reported that treatment with chemotherapy and radiotherapy in the management of a number of different cancers, among bladder cancers, are one of the major treatment modalities. However, the treatment of urothelial cancer with either chemotherapy and radiotherapy or a combination of both has been considered as a double-edged solution, since it has been reported earlier that bladder cancer patients treated with chemotherapy followed by radiotherapy appeared to have leukemogenic effect due to the fact that some of the components of these treatment strategies have been responsible for long-term bone marrow toxicit y[28]; moreover, additional treatment with radiotherapy to attain a complete response may participate in a part of toxicity directing to acute myeloid leukemia (AML) or induce cancer after treatment [29]. In the present cases, three patients were tested positive for a pathogenic somatic mutation in BRCA genes; two of them developed another type of malignancy which might be consequences for the chemo and/or radiotherapy. Thus, the FDA approved the number of PARP inhibitors drugs in breast, ovarian, and prostatic cancers for patients who test positive for pathogenic BRCA, so the same thing reflects the importance of BRCA testing in bladder cancer patients which might open new safer therapeutic strategy build on the ICB for those patients [30].

## Conclusion

According to our knowledge, this is the first analysis on paired tumor and blood analysis from bladder cancer patients on BRCA gene using NGS and shades the light on the mutation status of BRCA 1 and 2 clarifying that the tumor tissues showed somatic events which were not present in the germline DNA which reflects the importance of BRCA1and 2 somatic mutation testing and how it might affect the treatment strategy selection and predicting disease prognosis leading to better personalized medicine.

## Data Availability

Not applicable

## Abbreviations

BRCA: Breast cancer
FGFR3: Fibroblast growth factor receptor3
RB1: Retinoblastoma1
HRAS: Gene belong to RAS
TP53: Total p53
TSC1: Hamartin
H2AX: Histone A
dsDNA: Double stranded
TURBT: Transurethral resection of bladder tumor
NMIBC: Non-muscular invasive bladder cancer
TLC: Total leukocyte count
RCC: Renal cell carcinoma
CMD: Chronic medical diseases
TCC: Transitional cell carcinoma
EDTA: Ethylenediaminetetraacetic acid
DDR: DNA damage response
ICB: Immune checkpoint blockade
IFN: Interferon response
TILs: Tumor-infiltrating lymphocytes
*CCL5*: Chemokine (C-C motif) ligand 5
